# Lactation in Advanced Life

**Published:** 1874-07

**Authors:** T. S. Hopkins

**Affiliations:** Thomasville, Ga.


### LACTATION IN ADVANCED LIFE.

By T. S. HOPKINS, M.D., of Thomasville, Ga.

Whilst the following cases are far from being without a parallel, they are sufficiently uncommon to be considered worthy of publication. They are the only cases of the kind I have seen in a practice of thirty years.

Case 1. Maria Neil, colored, aged sixty years, lost a daughin child-bed in the fall of 1871, who left an infant six days old. Maria took the child home. When about seven months old she brought it to me to prescribe for with diarrhœa. Upon inquiry as to its diet she informed me that she was nursing it from her own bosom. The operation of nursing was gone through with in my presence, and was entirely satisfactory to the child. Before nursing the child, squeezing the gland would cause the milk to flow as in young women. Maria's youngest child was eighteen years of age when the infant was applied to her breast. The child is now about two years old, strong and in fine health. To quiet the child at night the old woman had placed her nipple in its mouth, and "by and by milk come," and still it comes.

Case 2. Letta Terry, colored, is nursing her grand-child. The mother died when the child was two months old. Letta gave the child her nipple. In about two months time the functions of the flabby, atrophied glands which had ceased for seventeen years, were restored, and milk flowed freely from them, a slight squeeze causing it to spirt for some distance. Letta's youngest child was married seventeen years ago. This child is not in good health; it is teething, and shows evidence of mal-nutrition. The anterior fontanel shows no disposition to close. It has never had diarrhœa, but is disposed to constipation. I regret that these old women, in whom the function of lactation has been restored, are *widows*, and likely to remain so, otherwise we might have an opportunity of testing the effect the restored mammary functions might have on the ovarian activity.

